# Case Report: Novel Homozygous Likely Pathogenic *SCN1A* Variant With Autosomal Recessive Inheritance and Review of the Literature

**DOI:** 10.3389/fneur.2021.784892

**Published:** 2021-11-30

**Authors:** Ana Victoria Marco Hernández, Miguel Tomás Vila, Alfonso Caro Llopis, Sandra Monfort, Francisco Martinez

**Affiliations:** ^1^Neuropediatrics Section, Hospital Universitari i Politècnic La Fe, Valencia, Spain; ^2^Genetics Unit, Hospital Universitari i Politècnic La Fe, Valencia, Spain; ^3^Genomics Unit, La Fe Health Research Institute, Valencia, Spain

**Keywords:** *SCN1A*, Dravet syndrome, GEFS+, homozygous, autosomal recessive inheritance

## Abstract

Dominant pathogenic variations in the *SCN1A* gene are associated with several neuro developmental disorders with or without epilepsy, including Dravet syndrome (DS). Conversely, there are few published cases with homozygous or compound heterozygous variations in the *SCN1A* gene. Here, we describe two siblings from a consanguineous pedigree with epilepsy phenotype compatible with genetic epilepsy with febrile seizures plus (GEFS+) associated with the homozygous likely pathogenic variant (NM_001165963.1): c.4513A > C (p.Lys1505Gln). Clinical and genetic data were compared to those of other 10 previously published patients with epilepsy and variants in compound heterozygosity or homozygosity in the *SCN1A* gene. Most patients (11/12) had missense variants. Patients in whom the variants were located at the cytoplasmic or the extracellular domains frequently presented a less severe phenotype than those in whom they are located at the pore-forming domains. Five of the patients (41.7%) meet clinical criteria for Dravet syndrome (DS), one of them associated acute encephalopathy. Other five patients (41.7%) had a phenotype of epilepsy with febrile seizures plus familial origin, while the two remaining (17%) presented focal epileptic seizures. *SCN1A-*related epilepsies present in most cases an autosomal dominant inheritance; however, there is growing evidence that some genetic variants only manifest clinical symptoms when they are present in both alleles, following an autosomal recessive inheritance.

## Introduction

*SCN1A* gene encodes the alpha 1 subunit of the sodium channel, Nav1.1, an important protein that makes up the voltage-dependent sodium channels. Pathogenic variants cause a reduction in sodium currents in gamma-aminobutyric acid (GABA)-ergic inhibitory interneurons, leading to hyperexcitability of neuronal network and the appearance of seizures (1–3). *SCN1A* is associated with several epilepsy syndromes and a range of other diseases (4, 5). The most common is Dravet syndrome (DS), but it is also associated with generalized epilepsy with febrile seizures plus (GEFS+), familial hemiplegic migraine (FHM), autism spectrum disorder (ASD), Sudden Unexpected Death Epilepsy (SUDEP), and epilepsy of infancy with migrating focal seizures (EIMFS) and very rarely involved in infantile spasms (4, 6–9). Genetic epilepsy with febrile seizure plus (GEFS+) is a familial epilepsy syndrome in which affected individuals within a family typically have a variety of epilepsy phenotypes, varying from simple febrile seizures and febrile seizures plus with a good outcome to severe epileptic encephalopathies (10).

About 1,871 *SCN1A* pathogenic variants have been identified so far (11), almost all of them under an autosomal-dominant fashion.

An attempt has been made to explain the phenotypic variability associated with *SCN1A* based on the type of mutation or functional alteration; in general, missense mutations are negatively correlated with a severe phenotype, and mutations with severe phenotypes are more frequently located in the pore region (12). Pathogenic variants that lead to a complete loss of function of the channel are virtually always associated with severe phenotypes, whereas milder impairments in channel function usually cause milder phenotypes (5, 12). Mutations that produce gain of function have also been described, as missense p.Thr226Met variant, which associates a severe clinical phenotype of early-onset epileptic encephalopathy, with profound developmental delay and hyperkinetic movement disorder (13, 14). However, in clinical practice, it remains difficult to fully predict the effects of novel variants on channel function. Recently, Jaber et al. have published original research reporting three patients with arthrogryposis multiplex congenita (AMC) in relation to other heterozygous missense variants of *SCN1A* (15).

To date, only 10 patients with *SCN1A*-related epilepsy due to autosomal recessive inheritance have been identified. In 2015, Brunklaus et al. published for the first time two novel homozygous missense mutations of the *SCN1A* gene in four children with infantile epilepsies from two consanguineous pedigrees. In both families, the heterozygous-carrier parents remained unaffected while their homozygous children developed *SCN1A*-related epilepsies, including DS and GEFS + (14). Since then, six more patients have been published (16–20). Here, we report two siblings, sons of a family with high consanguinity, with GEFS+ associated with the homozygous c.4513A > C variant in the SCN1A gene.

## Materials and Methods

### Participants

The study was carried out in patients affected by homozygous *SCN1A* pathogenic or likely pathogenic variants, according to the American College of Medical Genetics and Genomics criteria (21), from Hospital La Fe. One family was recruited for inclusion. Informed consent was obtained from the parents of both participants. A systematic search was carried out in the scientific literature for other patients diagnosed with *SCN1A*-related epilepsy with recessive inheritance. The search terms in Pubmed were “*SCN1A*,” “homozygous,” and “recessive inheritance.”

A brief clinical description of this cohort can be seen in Table 1.

**Table 1:** Clinical information on 12 patients with pathogenic or likely pathogenic *SCN1A* variants with autosomal recessive inheritance NM_001165963.1 (see Figure 2).

	**Patient 1** **4 y female**	**Patient 2** **35 mo male**	**Patient 3** **9 y male**	**Patient 4** **7 y female**	**Patient 5** **30 y male**	**Patient 6** **19 y female**
Variant	chr2:166852591T>G c.4513A>C (p.Lys1505Gln)		chr2:166915161C>T c.302G>A (p.Arg101Gln) chr2:166850781A>G c.4727T>C (p.Ile1576Thr)		c.2768T>G, p.(Ile923Ser) Homozigous	c.824A >G, p.(Asn275Ser) Homozigous
Medical history	None	None	None	None	None	None
Family background	Consanguineous (Figure 1) Mother's sister and first-counsin with S Second paternal uncle with S		Consanguineous Sister died at 2 months from SE Asymptomatic father, mosaic		Consanguineous Sister with similar phenotype, age at seizure onset 10 mo	Consanguineous (first cousins) Brother is patient 11
Age al seizure onset	4 mo	7 mo	3 mo	11 mo	19 mo	3 mo
Seizure type al onset	FS (20 min)	FS (3 min)	GTCS and PS	GTCS and PS		FS
Seizure types	FS, FSE, GTCS	FS, FSE (30 min)	MS, AS	FS, MS, AS	FS, HCS afebrile and febrile	FS, PS, AS, GTCS
Seizure triggers	Fever	Fever			Fever	Fever, LMT, CBZ
Medication trials **Actual medication**	FB, VPA. **LEV**	**LEV**			**LEV, ZNS** (others)	LMT, CBZ, LEV, TPM, CLB**, VPA**
Additional clinical features	None	Sensorineural deafness	Weight and height measurements *p* < 3			ADHD
Intellectual disability	No	No Hypotonia	Yes	Yes	No, learning difficulties, slow reasoning	Borderline,
Type of movement disorder/Neurologic examination	None/Normal	None/Normal			“Clumsy”	
Language disturbance	No	No				
Brain MRI	Normal	Normal	Mild asymmetric widening of the posterior horn of the left ventricle, slight non-specific white matter change in T2 weighted images	Normal	Normal	Normal
EEG	Normal	Normal		Generalized epileptiform activity with no distinctive photosensivity	Normal, minor fronto.temporal abnormalities	Normal, diffuse spike-waves in temporal and frontal
Phenotype	GEFS+	GEFS+	DS	DS	GEFS+	GEFS+
Reference	This work	This work	Tuncer et al. (17)		Moretti et al. (20)	
	Patient 7 14 y female	Patient 8 4.5 y male	Patient 9 17 y male	Patient 10 5 y female	Patient 11 8 y male	Patient 12 3 y male
	**Patient 7** **14 y female**	**Patient 8** **4.5 y male**	**Patient 9** **17 y male**	**Patient 10** **5 y female**	**Patient 11** **8 y male**	**Patient 12** **3 y female**
Variant	chr2:166903459T>C c.1198A>G (p.Met400Val)		chr2:166900370G>A c.1852C>T (p.Arg618Cys)		chr2:166856252G>A c.4319C>T (p.Ala1440Val)	chr2:166850711AT>A c.4796delA (p.Tyr1599Phefs*19)
Medical history			None		None	None
Family background	Parents are first-cousins Young brother with bilateral sensorineural hearing loss Maternal first cousin with febrile seizures until age five.		Parents are first-cousins Older sister with sensorineural hearing impairment due to connexin 26 mutation		Non-consanguineous parents	Both parents are heterozygous for this variation
Age al seizure onset	5 mo	15 mo	6 mo	13 mo	6 mo	4 mo
Seizure type al onset	FS 10 min	FS 20 min	Febrile GTCS 5 min	FS 10 min	FS	Afebrile left FMS
Seizure types	FS, Hemiclonic, GTCS, FSE, AA (atypical absences)	FSE, afebrile GTCS.	FS, Short afebrile focal seizures, night-time S frontal semiology	FS, PS with ASC characterized by behavioral arrest followed by head turning and hemi-clonic jerking	FS, GTCS, afebrile HCS, febrile HCS (5–15 min), MS (“head nodding,” and “jerking of the legs”), PS, SE (>30 min), multiple types S.	FMS, AAS
Seizure triggers	CBZ, fever	Fever	Fever	Fever	Fever	Fever
Medication trials Actual medication	CBZ	None	CBZ, LMT, TPM, LEV, STP, VPA, CZ	CBZ, VPA	VPA, LEV, TPM, BZD, FB, P	OXC, FTN, VPA, LEV, FB.**VPA, TPM, CZ**
Additional clinical features					AE: SE at 2y → F0E0 no eye contact	Hepatomegaly
Intellectual disability	Yes, profound learning disability	No	DMD Severe learning difficulties	5y No	Hyperactive behavior, DMD. After AE: Profound DMD, behavior problems autistic traits	Neuromotor developmental retardation
Type of movement disorder/Neurologic examination	Diskinetic movement disorder characterized by mixed spasticity and dystonia		Balance and motor coordination problems		Ataxia After AE: spastic quadriplegia	Ataxic walking without support. Hand stereotypic movements
Language disturbance					After AE: total loss of speech	Meaningless word repetitions
Brain MRI	20 mo: small corpus callosum with markedly delayed myelination and an AC	15 mo: normal	8 and 12 y: normal	4 y: normal	1 y: normal	Normal
EEG	19 mo: diffusely slow 11 y: awake: slow background of low amplitude.Sleep recurrent bursts of rhythmic sharp w. 14 y: interictal epileptiform activity	15 mo: normal		14 mo: normal 3 y: bilateral bursts of 3–4 Hz spike and wave. No photosensitivity	1 y: normal 21 mo: epileptic activity with slow waves and multifocal spike and spike-wave discharges which tended to increase with photic stimulation	4 mo: normal 3 y: frequent repetitive generalized spike-slow waves and multiple spike-slow wave activities
Phenotype	DS	FS+	FS+ PS	FS+ PS	DS and AE	DS
Reference	Brunklaus et al. (16)				Khanh Van et al. (19)	Aslan et al. (18)

*AAS, atypical absence seizure; AC, atrophic cerebellum; AE, Acute encephalopathy; AS, absence seizure; ASC, altered state of consciousness; CBZ, carbamazepine; CZ, clobazam; DS, Dravet syndrome; FSE, febrile status epilepticus; FB, fenobarbital; FS, febrile seizures; FTN, fenitoina; GEFS+, genetic epilepsy with febrile seizures plus; GTCS, Generalized tonic-clonic seizure; HCS, hemiclonic seizure; LEV, levetiracetam; LMT, lamotrigine; mo, months; MS, myoclonic seizures; OXC, oxcarbazepine; P, propofol; PS, partial seizure; S, seizure; STP, stiripentol; TPM, topiramato; VPA, valproate; Y, year; w, waves*.

This study was approved by the local ethics committee of the Hospital Universitari i Politècnic La Fe (Valencia, Spain).

### Genetic Studies

Peripheral blood samples from individuals and their parents were collected by standard methods for the genetic studies. Genomic DNA was isolated using the Qiacube extractor (QIAGEN, Hilden, Germany). DNA purity and concentration were measured using the NanoDrop 8000 spectrophotometer (Thermo Scientific) and a Qubit 2.0 fluorometer (Invitrogen), respectively.

Massive parallel sequencing for exome sequencing was performed by using the SureSelect Clinical Research Exome (Agilent Technologies). The libraries were sequenced on an Illumina NextSeq 500 following the manufacturer's protocol to get a mean reading depth of 100 X. Sequence read alignments, variant calling, and annotation were performed in the *Alissa Interpret* platform (Agilent Technologies). All disease-causing genes related with epilepsy and/or neurodevelopmental disorders described in different databases were analyzed. To evaluate the clinical impact and to assess the pathogenicity of variants in exome sequencing, we applied the previously described criteria (22). All clinically relevant genetic variants detected were confirmed by Sanger sequencing from purified PCR products. Primers for amplification and Sanger sequencing were designed with exon-primer (primers and PCR conditions are available on request).

## Case Studies

### Patient 1

**Figure 1:**
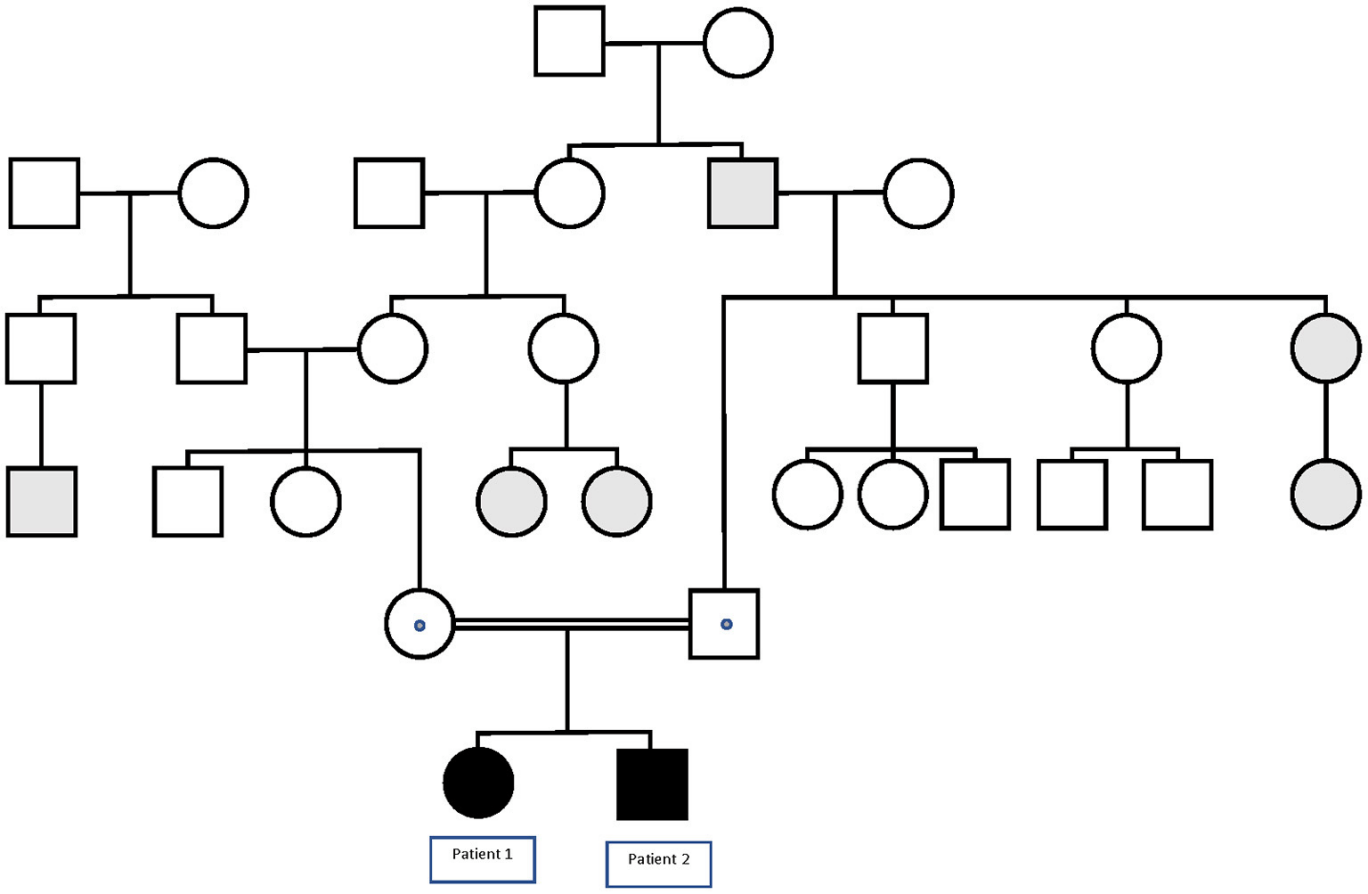
Pedigree of the family in which the p.Lys1505Gln variant in the *SCN1A* gene segregates with GEFS+. Black symbols denote homozygous patients, and dotted symbols denote obligate or confirmed heterozygous healthy carriers. Gray symbols denote patients with FS or FS+.

Patient 1 is a 4-year-old female born to healthy consanguineous parents (see Figure 1). During pregnancy, no antenatal problems were detected, neither any problem during delivery. The patient lived in her country of origin (Romania) for up to 12 months. The family reported that, at 4 months of age, she presented the first febrile status epilepticus (SE) (20 min). At 7 months, she presented a 10-min febrile seizure (FS), for which phenobarbital was prescribed. She presented two more FS up to 11 months. At 11 months, she presented first generalized tonic-clonic seizure (GTCS) lasting 5 min without fever. So, treatment with valproic acid was established. At 12 months, she was assessed for the first time in our center. She presented age-appropriate psychomotor development. Brain MRI and electroencephalogram study were normal. In the metabolic study, lactic acid, ammonium, and organic acids were normal. In the acylcarnitine profile, a slight decrease in free carnitine 11.95 μmol/L (12.92–36.88) was detected, probably secondary to treatment with valproate, so it was decided to suspend phenobarbital and valproic acid to start treatment with levetiracetam. Since then, she has presented four more FS in the context of infectious illness, and her psychomotor development and neurological examination are normal. The clinical phenotype of the patient is GEFS+.

### Patient 2

Patient 2 is a 35-month-old male, the brother of Patient 1 (see Figure 1). The child was born from uneventful eutocic delivery after a 37-week regular gestation, with a birth weight 3,580 g; Apgar score, 8/9/10. In the screening with otoacoustic emissions carried out at birth, hearing loss was detected, which was confirmed by performing auditory-evoked potentials of the trunk (response, 70 dB in both ears with dysmorphic curves). At 7 months of age, he presented the first FS (3 min). Furthermore, at that age, significant axial hypotonia was detected, without maintaining head support and little visual contact. For this reason, it was decided to perform an MRI study and electromyogram, which were normal. Metabolic study was extended to ammonium, amino acids in serum and cerebrospinal fluid, acylcarnitines, and organic acids, all in the normal range. At 10 months of age, hearing aids were placed, and early stimulation was started with very good evolution. In the following months, the patient presented a total of four more typical FS. At 20 months of life, he presented a febrile SE of 30 min in the context of high fever, so it was decided to start treatment with levetiracetam. Subsequently, he has only presented a more typical FS.

Currently, psychomotor development is at the lower limit of normality, and the neurological examination is normal. The clinical phenotype of the Patient 2 is compatible with GEFS+.

### Genetics

**Figure 2:**
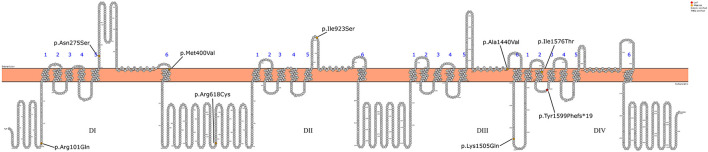
Schematic representation of the *SCN1A* gene mutations detected in the 12 patients.

Initially, Patient 2 was genetically studied due to hearing loss; only the heterozygous variant c.269T > C (p.Leu90Pro) in the *GJB2* gene was considered of relevance. The inheritance pattern of hearing loss due to this gene is autosomal recessive and requires the presence of a second mutation in this gene to confirm the genetic diagnosis of hearing loss. It is possible that there might be a second undetected mutation or that there is another genetic or non-genetic cause of the hearing loss. On the other hand, Patient 2 presents the homozygous variant c.4513A > C in the *SCN1A* gene as the only clinically relevant result. This variant causes the substitution of the highly conserved amino acid lysine by glutamine in position 1,505 of the protein (p.Lys1505Gln), predicted as likely pathogenic by most of the *in silico* predictors (see Table 2). This variant has not been previously described. The variant is located between the repeat Domains III and IV at the cytoplasmic domain (see Figure 2). According to the ACMG criteria, this variant was classified as likely pathogenic (PP3, PM1, PM2, and PP2).

**Table 2:** Genetic information on 12 patients with pathogenic or likely pathogenic *SCN1A* variants with autosomal recessive inheritance NM_001165963.1.

**Patient**	**Patient 1 and 2**	**Patient 3 and 4**	**Patient 5**	**Patient 6**	**Patient 7 and 8**	**Patient 9 and 10**	**Patient 11**	**Patient 12**
Variant	chr2:166852591 T>G c.4513A>C (p.Lys1505Gln)	chr2:166915161 C>T c.302G>A (p.Arg101Gln) chr2:166850781 A>G c.4727T>C (p.Ile1576Thr)	chr2:166894464 A>C c.2768T>G (p.Ile923Ser)	Chr2: 166908369 T>C c.824A>G (p.Asn275Ser)	chr2:166903459 T>C c.1198A>G (p.Met400Val)	chr2:166900370 G>A c.1852C>T (p.Arg618Cys)	chr2:166856252 G>A c.4319C>T (p.Ala1440Val)	chr2:166850711 AT>A c.4796delA (p.Tyr1599Phefs*19)
Classification	Likely pathogenic	Pathogenic Likely pathogenic	Uncertain significance	Likely pathogenic	Pathogenic	Uncertain significance/Likely pathogenic	Likely pathogenic	Pathogenic
Inheritance	Inherited homozygous (from both parents)	Compound heterozygous	Homozygous		Inherited homozygous (from both parents)	Inherited homozygous (from both parents)	Homozygous *de novo*	Inherited homozygous (from both parents)
ACMG criteria	PM1 moderate, PM2 moderate, PP2 supporting, PP3 supporting	PM5 very strong, PM1 moderate, PM2 moderate, PM5 moderate, PP2 supporting, PP3 supporting, PP5 supporting PM1 moderate, PM2 moderate, PP2 supporting, PP3 supporting	PM2 moderate, PP2 supporting, PP3 supporting	PM1 moderate, PM2 moderate, PP2 supporting, PP3 supporting	PM1 strong, PM2 moderate, PM5 moderate, PP2 supporting, PP3 supporting, PP5 supporting	PM2 moderate, PP2 supporting, PP3 supporting	PM1 moderate, PM2 moderate, PP2 supporting, PP3 supporting	PVS1 very strong, PM2 moderate, PP3 supporting
CADD	28.6	29.6/26.9	26.9	23.1	23.7	29.6	33	33
Phenotype	GEFS+ (both)	DS (both)	GEFS+	GEFS+	DS (p7) FS (p8)	FS+ PS (both)	DS and AE	DS
Reference	This work	Tuncer et al. (17)	Moretti et al. (20)		Brunklaus et al. (16)		Khanh et al. (19)	Aslan et al. (18)

Familial segregational study by Sanger sequencing confirmed that Patient 1 shares the variant c.4513A > C in *SCN1A* gene in homozygous state, while the healthy parents of the two patients were heterozygous carriers.

## Discussion

So far, thousands of patients with heterozygous variants in the *SCN1A* gene have been published. The spectrum of the clinical phenotype associated with variations in the *SCN1A* gene is wide. Numerous studies have attempted to correlate the clinical phenotype according to the genotype (5, 12, 23), and there is evidence that, depending on the type and location of the mutation in the gene, the severity of the clinical picture varies. However, predicting the clinical consequences of novel genetic variants found in routine clinical practice is not an easy task. On the other hand, new phenotypes associated with *SCN1A* are continually being published (13, 15, 24–27).

The majority of published patients with pathogenic variants in *SCN1A* have autosomal dominant inheritance, more frequently by *de novo* mutation, although there are also many inherited cases (28). However, in recent years, several individuals who presented epilepsy in relation to homozygous or compound heterozygous variants in *SCN1A*, that is, with autosomal recessive inheritance, have been published. We have collected all the published cases and added our patients in order to broaden the phenotypic and genotypic spectrum associated with *SCN1A*.

In 2015, Brunklaus et al. published for the first time two novel homozygous missense mutations of the *SCN1A* gene in four children. Then, Tuncer et al. published two brothers with compound heterozygosity in *SCN1A* with symptoms compatible with DS (17). Subsequently, Aslan et al. and Van et al. have published two more cases with homozygous variants in patients with DS (18, 19), and Moretti et al. published two novel homozygous missense variations of the *SCN1A* gene in two individuals from two different families from consanguineous pedigrees. Both of them encompassed with GEFS+ (20) (see Tables 1, 2).

Ten of the total 12 patients were children of families with consanguinity. Patient 11, published by Van et al. presented the variant p.Ala1440Val in homozygosity, but the authors verified that it was a *de novo* mutation, since, despite having verified the paternity of the parents, none of them were carriers (19).

Regarding the clinical phenotype, five of the patients (41.7%) are clinically encompassed by Dravet syndrome (DS), one of them associated acute encephalopathy (AE). Five (41.7%) had a phenotype of GEFS+ of familial origin. Two (16.7%) of the patients presented FS+ and focal epileptic seizures (10). In Table 1, we have collected the description of all the patients.

Patient 2 presented sensorineural deafness, of unknown etiology, despite having carried out an extensive etiological study. Furthermore, Patients 7 and 8 had a younger brother with sensorineural hearing loss without a specified cause, and Patients 9 and 10 had an older sister with sensorineural hearing impairment due to connexin 26 mutation. Therefore, of the eight families in the series, three of them had individuals with sensorineural deafness. There are no publications linking *SCN1A* variants with sensorineural deafness, despite the large number of published patients with variants in this gene. For this reason, we think that this finding is probably related to consanguinity of the family rather than to *SCN1A* gene. The concurrence in a family of two autosomal recessive inherited diseases is not so uncommon when it comes to consanguineous families (29). Sensorineural deafness of genetic cause is mostly (about 80%) due to genetic variants of autosomal recessive inheritance (30, 31).

In the same way, we think that, possibly, the hepatomegaly that Patient 12 presented is unrelated to the variants in *SCN1A* gene.

Missense variants in *SCN1A* gene have variable effects, depending on the functional regions in which they occur and the nature of amino acid substitutions (11).

Missense mutations leading to disease are more likely to occur in the voltage sensor (S4) and the pore region (S5-S6 segments) of the four homologous domains, as in the case of Patients 3, 4, 7, and 11 of this series (32). As an exception, Patient 8 (the brother of Patient 7) presents the variant in the pore and a mild phenotype. In some pairs of siblings, a marked heterogeneity of the phenotype is observed. It has been proposed that variations in other epilepsy genes can modify the clinical phenotype associated with variants in *SCN1A* (33). This phenomenon has also been described in other genetic epilepsies associated with other ion channels, such as *SCN2A* (34). This variability associated with the same variation in *SCN1A* has been described both in individuals from the same family and at the interfamily level. However, further unknown environmental or epigenetic factors have been also proposed to play a significant role in the development and evolution of the epilepsy phenotype (35, 36).

All the variants described in the series are missenses, except for the frames hit variant in Patient 12, who presents a severe DS phenotype even though it is located at the intracytoplasmic level. So, due to the characteristics of the variant, the effect on the protein can be variable. Pathogenic variants that lead to a complete loss of function of the channel are virtually always associated with severe phenotypes.

It is difficult to establish a correlation between the genotype phenotype with such a small series; however, the data suggest that the variants located at the intracytoplasmic or extracellular level (Patients 1, 2, 5, 6, 9, 10, and 12) present a less severe epileptic phenotype. Conversely, those variants found at the pore region (Patients 3, 4, 7, 8, and 11) more frequently present a more severe phenotype, with a phenotype of Dravet syndrome in most patients.

It is worth noting that heterozygous carrier parents or other relatives are asymptomatic. Therefore, the variants described in this work are probably hypomorphic, both due to their location within the gene and because of their intrinsic properties, and they are only capable of altering the function of the Nav1.1 protein when they are in homozygous state. In any case, it is also remarkable that the phenotypic spectrum of the whole series of patients presenting the autosomal recessive phenotype related to SCN1A is quite similar to the phenotypic spectrum of most patients with autosomal dominant inheritance, as well as a similar genotype-phenotype correlation.

## Conclusions

The 12 patients published so far with an autosomal recessive epileptic phenotype associated with *SCN1A* show that not all patients with symptoms associated with this gene have autosomal dominant inheritance. On the other hand, it is worth noting that the resulting phenotypes do not differ from those usually caused by dominant heterozygous variants. Furthermore, the analysis of the variants found in each patient, correlating it with the phenotype he or she presents, allows us to know a little better the correlation of genotype-phenotype associated with *SCN1A*. It is possible that hypomorphic heterozygous variants only manifest when affecting the two alleles of this gene.

## Data Availability Statement

The datasets presented in this article are not readily available due to ethical and privacy restrictions. Requests to access the datasets should be directed to the corresponding author.

## Ethics Statement

The studies involving human participants were reviewed and approved by Comité de ética del Instituto de Investigación Sanitaria La Fe. Written informed consent to participate in this study was provided by the participants' legal guardian/next of kin. Written informed consent was obtained from the minor(s)' legal guardian/next of kin for the publication of any potentially identifiable images or data included in this article.

## Author Contributions

AM: drafting of the article, clinical examination of the patients, and bibliographic search. AC and SM: substantial contributions to analysis and interpretation of genetic data. MT: revising the article for important intellectual content and contribution to clinical data analysis. FM: analysis and interpretation of genetic data and revising of the article and final approval. All authors contributed to the article and approved the submitted version.

## Funding

This work had received financial support from the CM19/00181 Grant from the Carlos III Institute (AM) and the Mutua Madrileña Foundation (holded by SM). AC was supported by a research grant by Fundación Mutua Madrileña. Support for open access publication was provided by Fundación Síndrome de Dravet (Dravet Syndrome Foundation Spain) (grant number FSD-OASP-XI-III).

## Conflict of Interest

The authors declare that the research was conducted in the absence of any commercial or financial relationships that could be construed as a potential conflict of interest.

## Publisher's Note

All claims expressed in this article are solely those of the authors and do not necessarily represent those of their affiliated organizations, or those of the publisher, the editors and the reviewers. Any product that may be evaluated in this article, or claim that may be made by its manufacturer, is not guaranteed or endorsed by the publisher.
